# Susceptibility of female rats to cardiac arrhythmias following refeeding after severe food restriction

**DOI:** 10.1186/s13293-022-00419-1

**Published:** 2022-03-15

**Authors:** Aline M. A. De Souza, Jonathas F. Q. Almeida, Nataliia Shults, Hong Ji, James Li, Kathryn Sandberg

**Affiliations:** 1 Department of Medicine, Georgetown University, Suite 232 Building D, 4000 Reservoir Road, NW, Washington, DC 20057 USA; 2 Department of Internal Medicine/Cardiology, Rush University, Chicago, IL 60612 USA; 3 Department of Pharmacology & Physiology, Georgetown University, Washington, DC USA; 4 Department of Biostatistics, Bioinformatics and Biomathematics, Georgetown University, Washington, DC USA

**Keywords:** Langendorff, Vascular reactivity, Inadequate food intake

## Abstract

**Background:**

Many studies have shown malnutrition and inadequate caloric consumption have adverse acute effects on cardiovascular structure and function.

**Methods:**

To determine the adverse long term cardiovascular effects, we studied cardiac morphology and function in female (F) and male (M) severe food restricted rats 3 months after refeeding (sFR-Refed).

**Results:**

Two weeks of a normal chow diet in which calories were reduced by 60% decreased body weight (BW) by approximately 15% in both sexes. Within 2 weeks of refeeding, no differences in BW were detected between CT and sFR-Refed groups. However, male rats gained almost 3 times more BW than the females over the 3-month refeeding period. Sex differences were also observed in cardiac pathology. Hearts from F-sFR-Refed rats exhibited more atrophy and less hypertrophy, while M-sFR-Refed rats predominantly exhibited hypertrophic remodeling. While there were no differences in the frequency of ventricular arrhythmias induced by ischemia/reperfusion (I/R) in the isolated heart between M-CT and M-sFR-Refed rats, I/R induced twice as many arrhythmias in the F-sFR-Refed rats compared to F-CT.

**Conclusions:**

These findings indicate the female heart is more susceptible to the long term adverse cardiovascular effects of sFR months after refeeding. Thus, this study provides a rationale for studying sex differences in cardiovascular risk in individuals who experience sFR for voluntary (e.g., very low-calorie dieting) or involuntary (e.g., poverty) reasons earlier in life.

## Highlights

- What are the long-term effects of a 2-week period of severe food restriction (sFR) on cardiac structure and function months after refeeding (sFR-Refed) in male and female rats?
- This study shows sex differences exist in cardiac pathology months after refeeding. A majority of cardiomyocytes were atrophied in F-sFR-Refed rats, while in M-sFR-Refed rats, the cardiomyocytes predominantly exhibited hypertrophic remodeling.
- While there were no differences in the frequency of ventricular arrhythmias induced by ischemia/reperfusion (I/R) in the isolated heart between M-CT and M-sFR-Refed rats, I/R induced twice as many arrhythmias in the F-sFR-Refed rats compared to the controls.
- Our findings have implications for the long-term risk of developing cardiovascular disease in individuals who have voluntarily or involuntarily experienced periods of sFR earlier in their lives, and that woman may be at greater cardiovascular risk than men.

## Introduction

Inadequate caloric intake in the presence of malnutrition has adverse physiological effects on the cardiovascular system [1]. We previously developed a model of severe food restriction in rats in which the rate and duration of body weight (BW) loss is similar to what occurs in many individuals who engage in limited very low calorie dieting (< 1000 cal/day for ≥ 2 weeks) [2]. In this model, rats are maintained for 2 weeks on a severe caloric restricted (sFR) diet, in which normal daily caloric intake is reduced by 60%. Both female [3] and male [4] rats lose approximately 15% of their BW under these dietary conditions. At the end of the 2-week period, the sFR rats exhibit hypotension and bradycardia, which is also observed in people experiencing similar degrees of caloric restriction [3, 5].

More recently, we showed that isolated hearts from M-sFR rats exhibited more cardiac pathology including myofibrillar disarray with contractions and cardiomyocyte lysis than hearts from the control group (M-CT) [4]. Not only did the M-sFR hearts exhibit cardiac pathology, they were also more susceptible to ventricular arrhythmias induced by ischemia/reperfusion (I/R). Isolated hearts from the M-sFR rats had a 1.7-fold increase in the frequency of cardiac arrhythmias in response to I/R compared to the M-CT rats.

Less is known about the long-term effects of sFR on cardiovascular function months after BW is restored due to refeeding. Therefore, in this study, we investigated heart morphology and function in female and male sFR rats 3 months after refeeding (sFR-Refed).

## Methods

### Ethical approval

All procedures were conducted in accordance with the NIH guide for the Care and Use of Laboratory Animals and Animal Research and the Reporting of in vivo Experiments (ARRIVE) guidelines. All procedures were also approved by the Georgetown University Animal Care and Use Committee.

### Animals

All experiments were conducted in female and male Fischer 344 rats bought from Envigo Corp. (Frederick, MD) when they were 12 weeks. All rats were single housed 1 week prior to, during and 1 week after the sFR period. For the remainder of the experiments, the rats were housed 2 per cage. All rats were maintained in a temperature-controlled facility at 24 °C on a 12-h light–dark cycle.

### Diet

All rats were maintained on a normal rodent diet (Rodent diet 20, #5053; LabDiet, Marlborough, MA). The amount of food provided to the sFR groups was calculated individually for each rat and was based on 40% of their average food intake during the week prior to initiation of the sFR period. After the 2-week sFR period ended, all rats were provided food ad libitum for the following 3 months. Water was provided to all rats ad libitum for the duration of the experiment.

### Food and water intake

During the week prior to and during the sFR period, food intake was determined every day between 5 pm and 6 pm in both the CT and sFR groups. After the sFR period ended, food intake was measured weekly at the same time of day in all rats. Water intake was measured twice a week at this same time of day for the duration of the study in all rats.

### Tissue size and mass

Immediately after the I/R experiment was concluded, hearts were weighed and the tibia length was measured using a caliper ruler.

### Cardiac histology and morphology

After the isolated heart experiment, cardiac tissue was fixed for histology and morphology as we previously described [4]. In brief, fixed tissue sections were subjected to hematoxylin/eosin (H&E) and Masson’s trichrome staining*.* Morphometric analysis was performed under blinded conditions (by NS) using systematic uniform random sampling with the Fiji software [6] version ImageJ2 [7] on 25 randomly selected images. The thickness of single cardiac myocytes was measured on H&E stained myocardium images of the left ventricle (LV). For quantifications of collagen deposition in the LV, the percent of fibrosis was calculated using morphometric grid images from Masson’s trichrome-stained slides [8].

#### Cardiac function in isolated heart

At the end of the 3-month refeeding period, cardiac function was assessed in the Langendorff isolated heart perfusion system as we previously described [4]. In brief, isovolumetric recording was determined with a small fluid-filled balloon inserted into the left ventricle through the left atrium, which was connected to a transducer coupled to a data-acquisition system (PowerLab, ADInstruments, Colorado Springs, CO). Coronary perfusion pressure was measured using a similar pressure transducer listed above connected to an aortic cannula. The perfusion flow was maintained at a constant rate of 10 ml/min and the balloon was inflated until the left ventricular diastolic pressure reached 10–15 mmHg. The balloon was handmade as described by Sutherland et al. [9] and the size was adjusted for each sex based on pilot tests; female: 3 × 3 mm, male: 4 × 4 mm. Before starting the experiment, the transducers were calibrated using a sphygmomanometer and the balloons were tested for possible leaking. Diastolic pressure was controlled during the baseline period to range between 10 and 15 mmHg before the ischemic period began by controlling the balloon inflation level and by the addition of a small drain on the base of the left ventricle.

#### Cardiac arrhythmias in isolated heart

The frequency of cardiac arrhythmias induced by I/R in the isolated heart was determined as we previously described [4]. In brief, after 20 min of habituation and 10 min of baseline data recording, global ischemia was induced by occlusion of inflow lines for 30 min. The heart was then reperfused for 60 min. The incidence of left ventricular arrhythmias during the reperfusion period was manually counted and expressed as a percentage of the total reperfusion time (i.e., the percent of time in which arrhythmias were detected during the entire perfusion period).

### Statistical analysis

Statistical comparisons were assessed using GraphPad Prism software (version 9, GraphPad Inc., La Jolla, CA). The effect of diet and sex were analyzed by two-way ANOVA (repeated measures when appropriate) followed by Bonferroni post hoc test. Data are expressed as means ± standard error of the means. Significance was defined as *p* < 0.05. The Gaussian kernel density estimates for univariate observations were used to analyze cardiomyocyte thickness from H&E-stained hearts in male and female CT and sFR-Refed groups. Estimates were computed by the density function in the R statistical program (version 3.6.1 by R-core, R-core@R-project.org). The algorithm disperses the mass of the empirical distribution function over a regular grid of at least 512 points and then uses fast Fourier transform to convolve this approximation with a discretized version of the kernel and then uses linear approximation to evaluate the density at the specified points. The statistical properties of a kernel were determined by ${\sigma }_{K}^{2}=\int {t}^{2}K(t)\mathrm{d}t$, which is always 1, and $R(K)=\int {K}^{2}(t)\mathrm{d}t$. Mean squared error-equivalent bandwidths (for different kernels) are proportional to ${\sigma }^{K}R(K)$, which is scale invariant and for our kernels equal to $R(K)$. Unequal variance based on two sample *t* tests were used to test the significance between groups.

## Results

### Effect of refeeding after severe food restriction on body weight

**Fig. 1:**
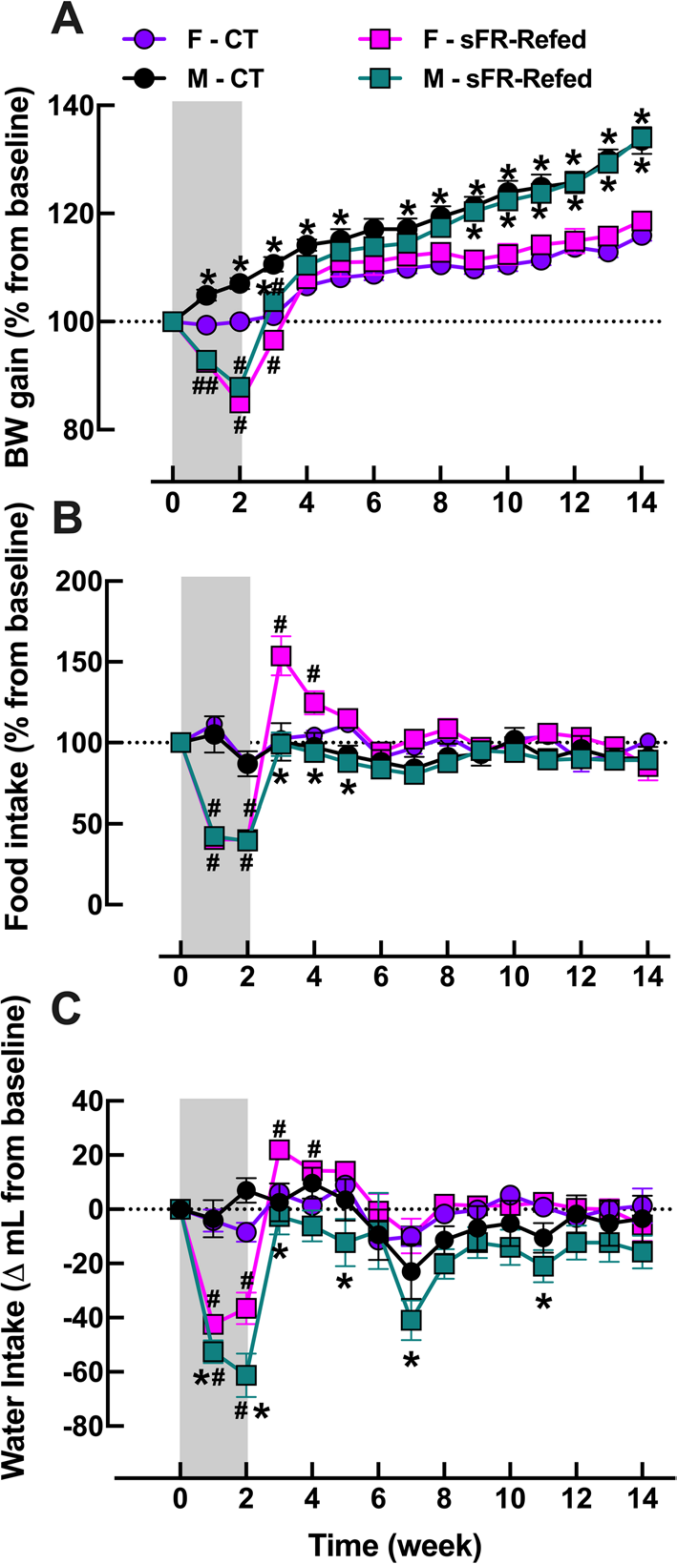
Effect of 3 months refeeding after severe food restriction on body weight gain and food intake. Change in **A** body weight (BW) and **B** food and **C** water intake compared to baseline in female (F, purple circle, red square; *n* = 9/group) and male (M, black circle, green square; *n* = 8/group) rats maintained on a normal diet for the duration of the study (CT, circle) or for 3 months after a 2-week severe food restricted period (sFR-Refed, square). Values are expressed as the mean ± SEM; ^#^*p* < 0.05 vs. CT, same timepoint, same sex and *p < 0.05 vs. F, same diet, same timepoint by two-way ANOVA and Bonferroni post-hoc test

Before beginning the sFR protocol, there were no differences in initial BW between the CT and sFR groups within each sex (Table 1); however, the male rats were 1.6-fold heavier than the female rats. BW in the F-sFR and M-sFR groups rapidly dropped after initiation of the sFR period. By day 14, the sFR rats lost 13–16% of their initial BWs (Fig. 1A). In comparison, F-CT rats maintained their BW during this 2-week period, while the M-CT group gained BW [(Δ*g*): F-CT: 0.0 ± 2 vs. M-CT: 20 ± 5 vs. *p* < 0.05; *n* = 8–9). Three months after refeeding, no differences in BW were observed within each sex between the CT and sFR-Refed rats; however, the male rats gained 3 times more BW during this 3-month period than compared to the female rats (Table 1).

**Table 1:** Effect of 3 months refeeding after severe food restriction on body weight

Animal group	Initial BW, g (*n*)	Final BW, g (*n*)	ΔBW, g (*n*)
F-CT	178 ± 1.3 (9)	207 ± 2.6^†^ (9)	29 ± 1.8 (9)
M-CT	290 ± 4.1* (8)	387 ± 5.1*^†^ (8)	97 ± 6.2 (8)
F-sFR-Refed	178 ± 2.4 (9)	211 ± 2.8^†^ (9)	33 ± 2.5 (9)
M-sFR-Refed	288 ± 4.3* (8)	386 ± 5.7*^†^ (8)	98 ± 2.7 (8)

Shown is the initial and final body weight (BW) in male (M) and female (F) rats maintained on a normal diet for the duration of the study (CT) or for 3 months after a 2-week severe food restricted period (sFR-Refed). Values are expressed as the mean ± SEM; *n*, group size; *p < 0.05 vs. F, same diet, same timepoint and ^†^*p* < 0.05 vs. initial timepoint, same sex by paired Student’s *t* test

### Effect of refeeding after severe food restriction on food intake

For the duration of the 2-week sFR period, daily food intake in the F-sFR and M-sFR groups was 40% of the F-CT and M-CT groups, respectively (Fig. 1B). During the 1st week of refeeding, the F-sFR-Refed rats ate 54% percent more than the F-CT rats; however, the increased food intake rapidly subsided to F-CT levels by week 5. In contrast, the M-sFR-Refed rats did not increase their food intake throughout the refeeding period in comparison to the M-CT group (Fig. 1B).

### Effect of refeeding after severe food restriction on water intake

Average water intake in the F-sFR and M-sFR groups for the duration of the 2-week sFR period was 23% and 34% lower than the F-CT and M-CT groups, respectively (Fig. 1C). During the 1st week of refeeding, the F-sFR-Refed rats drank 15% more than the F-CT rats; however, the increased water intake rapidly subsided to F-CT levels by week 5. In contrast, the M-sFR-Refed rats did not increase their water intake throughout the refeeding period in comparison to the M-CT group (Fig. 1C).

### Effect of refeeding after severe food restriction on heart weight, heart mass index (HMI) and tibial length

Three months after refeeding, no differences in the heart weight, HMI and tibial length were observed within each sex between the CT and sFR-Refed rats; however, the heart weight and HMI were approximately 2.3-fold higher in male compared to female rats, whereas minimal sex differences were observed in the tibia length (Table 2).

**Table 2:** Effect of refeeding after severe food restriction on tissue size

Animal group	Heart, g (*n*)	HMI, g/cm (*n*)	Tibia, cm (*n*)
F-CT	0.6 ± 0.01 (6)	0.147 ± 0.004 (6)	3.83 ± 0.076 (6)
M-CT	1.4 ± 0.04* (8)	0.329 ± 0.010* (8)	4.13 ± 0.018* (8)
F-sFR-Refed	0.6 ± 0.01 (5)	0.143 ± 0.002 (5)	3.96 ± 0.081 (5)
M-sFR-Refed	1.4 ± 0.05* (8)	0.344 ± 0.011* (8)	4.12 ± 0.018* (8)

Shown is the heart weight, heart mass index (HMI) and tibia length in male (M) and female (F) rats maintained on a normal diet for the duration of the study (CT) or for 3 months after a 2-week severe food restricted period (sFR-Refed). Values are expressed as the mean ± SEM; *n*, group size; *p < 0.05 vs. F, same diet by unpaired Student’s *t* test

### Effect of refeeding after severe food restriction on cardiac morphology

**Fig. 2:**
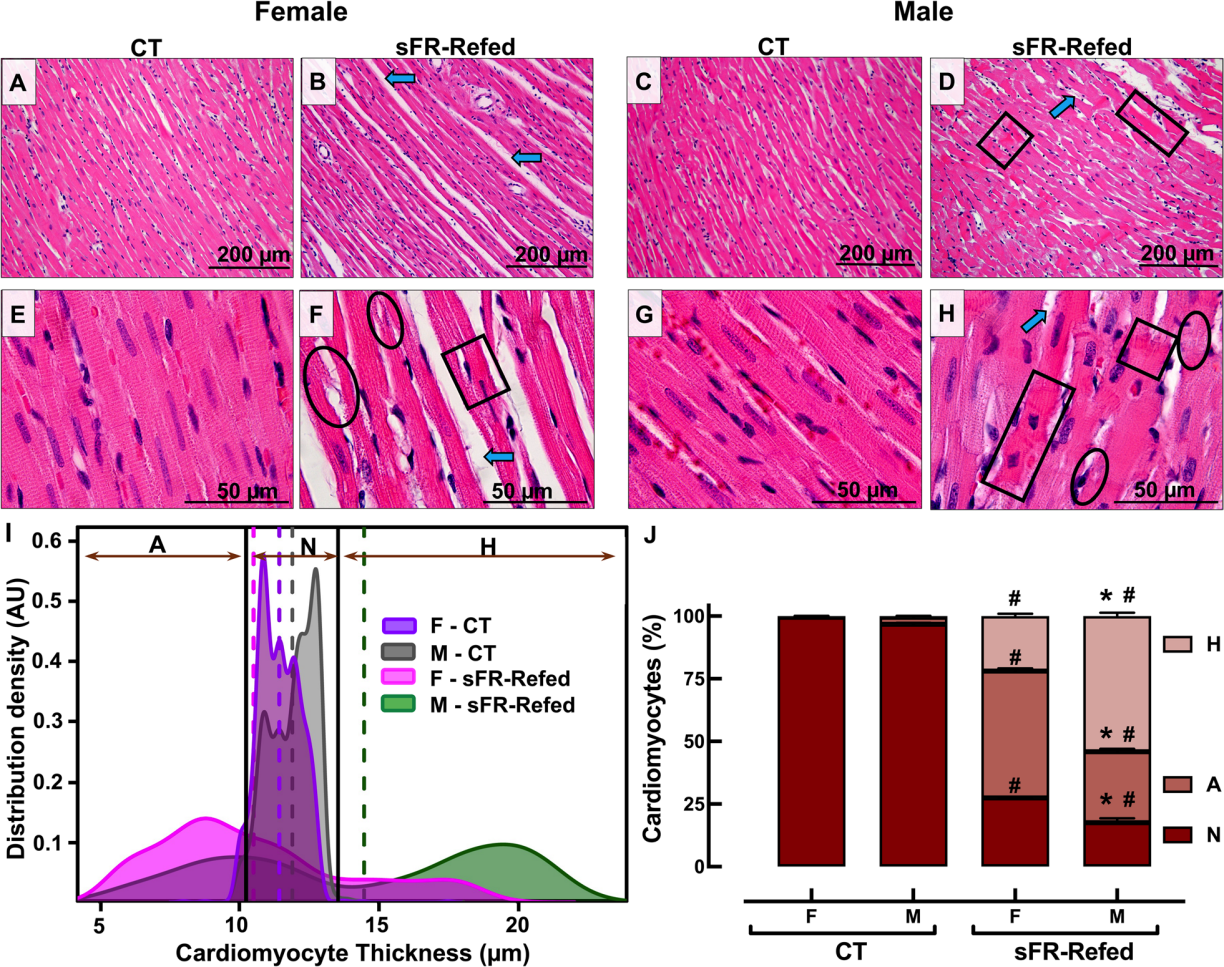
Effect of 3 months refeeding after severe food restriction on cardiac pathology in the isolated heart. **A**–**H** Representative sections of the left ventricle of the heart after staining with hematoxylin and eosin (H&E) in female (F) and male (M) rats maintained on a normal diet for the duration of the study (CT) or for 3 months after a 2-week severe food restricted period (sFR-Refed) at 200× (**A**–**D**) and 1000× (**E**, **F**) magnification. Images are representative of five rats per group. Grater interstitial space (blue arrow) is observed in F—compared to M-sFRRefed rats, while fiber constrictions (black box) and focal cell lysis (black circle) are more prevalent in M-sFR-Refed rats. **I** Gaussian Kernel density estimates of cardiomyocyte thickness from H&Estained hearts. The color coded vertical dashed lines represent the mean in each animal group. **J** The percentage of cardiomyocytes below (A, atrophied) and above (H, hypertrophied) the cardiomyocyte diameter normal (N) range (depicted by two black vertical lines). Values are expressed as the mean ± SEM; ^#^*p* < 0.05 vs. CT, same sex and *p < 0.05 vs. F, same diet by two-way ANOVA and Bonferroni post-hoc test

No sex differences were observed in cardiac morphology between CT-F (Fig. 2A, E) and CT-M (Fig. 2C, G) rats in H&E stained sections of the LV. Both F-CT and M-CT groups showed a typical pattern of parallel-orientated myocardial fibers and cross striation of cardiac myocytes in the LV. Out of an average of 220 cardiomyocytes counted per rat, the majority of female and male cells were within the normal size distribution [mean thickness (µm); F-CT 11.5 ± 0.75 vs. M-CT 11.9 ± 0.82; *p* = 0.73; *n* = 5/group] (Fig. 2I, J).

In contrast, hearts from F-sFR-Refed (Fig. 2B, F) and M-sFR-Refed (Fig. 2D, H) rats both showed disorganized myocardial fibers at 200× magnification (Fig. 2B, D). Under higher magnification (×1000), multiple contractile lesions and focal lysis of cardiomyocytes were observed (Fig. 2F, H). Furthermore, the majority of cardiomyocytes in both sexes were not within the normal Gaussian distribution of cardiomyocyte thickness (Fig. 2I, J). Only 27% and 17% of cardiomyocytes from the F-sFR-Refed and M-sFR-Refed groups, respectively, had cell thicknesses within two standard deviations of the mean of the CT cardiomyocytes. There were also significant sex differences in the Gaussian distribution of cardiomyocyte size. There was far more atrophy than hypertrophy in cardiomyocytes from F-sFR-Refed rats, whereas the opposite was true for cardiomyocytes from the M-sR-Refed rats (Fig. 2I, J).

### Effect of refeeding after severe food restriction on cardiac collagen deposition

**Fig. 3:**
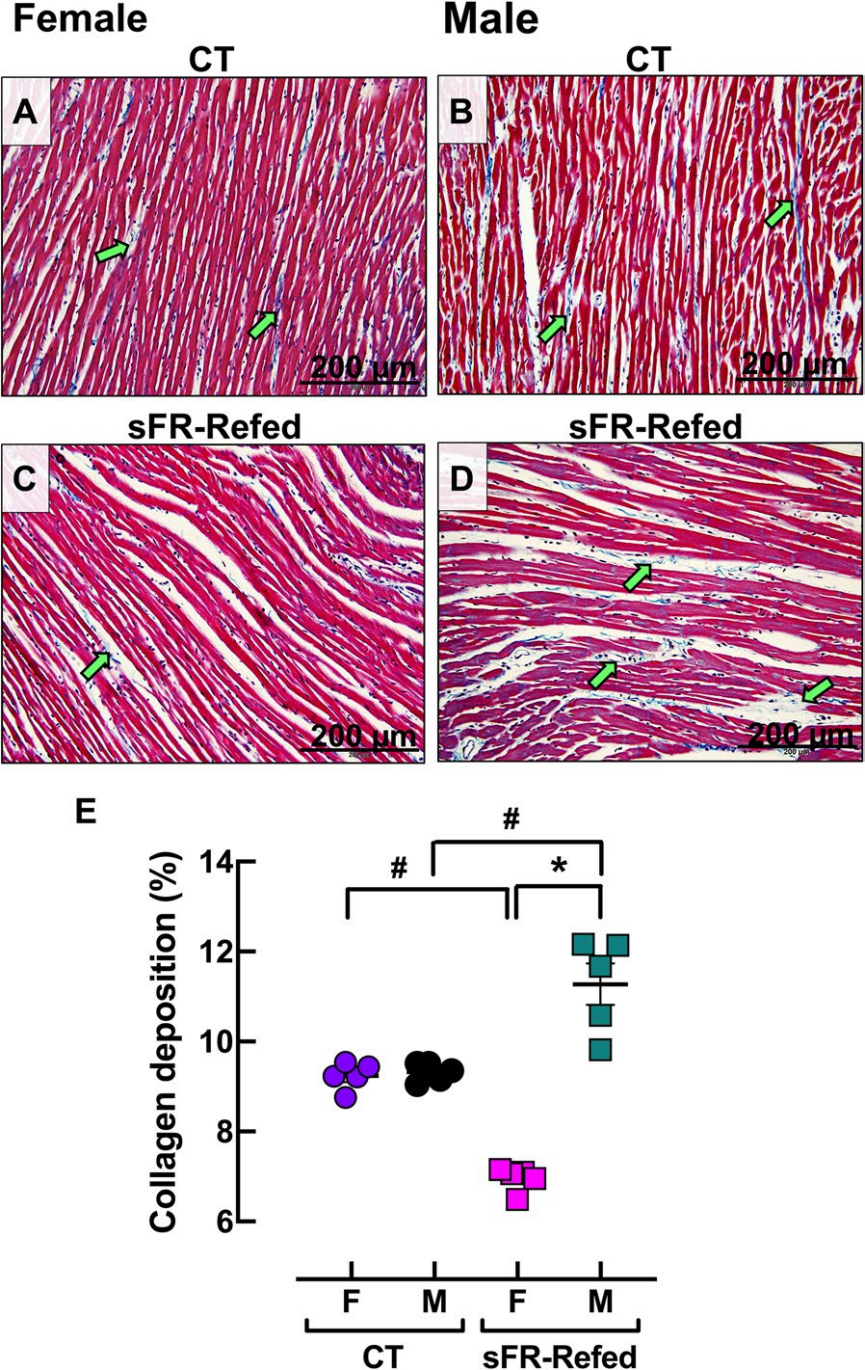
Effect of 3 months refeeding after severe food restriction on collagen deposition in the isolated heart. **A**–**D** Representative sections of the left ventricle of the heart after staining with Masson’s trichrome in female (**A** and **C**) and male (**B** and **D**) rats maintained on a normal diet for the duration of the study (CT) or for 3 months after a 2-week severe food restricted period (sFR-Refed) at 200-× magnification. Images are representative of five rats per group. More collagen deposition (green arrows) is observed in male (M)—compared to female (F)-sFR-Refed rats. **E** Quantitation of collagen deposition in CT and sFR-Refed rats in the hearts of both sexes. Values are expressed as the mean ± SEM; ^#^*p* < 0.05 vs. CT, same sex and *p < 0.05 vs. F, same diet by two-way ANOVA and Bonferroni post-hoc test

Morphometric analysis of Masson’s trichrome staining demonstrated no sex differences in collagen deposition in the LV from CT-F (Fig. 3A, E) and CT-M (Fig. 3B, E) rats. In contrast, the myocardium in F-sFR-Refed (Fig. 3C, E) and M-sFR-Refed (Fig. 3D, E) rats exhibited discordant changes in collagen deposition; the collagen deposition in the sFR-Refed rats was reduced in females, whereas it was increased in the males.

### Effect of ischemia/reperfusion on cardiac morphology in CT and sFR-Refed rats

**Fig. 4:**
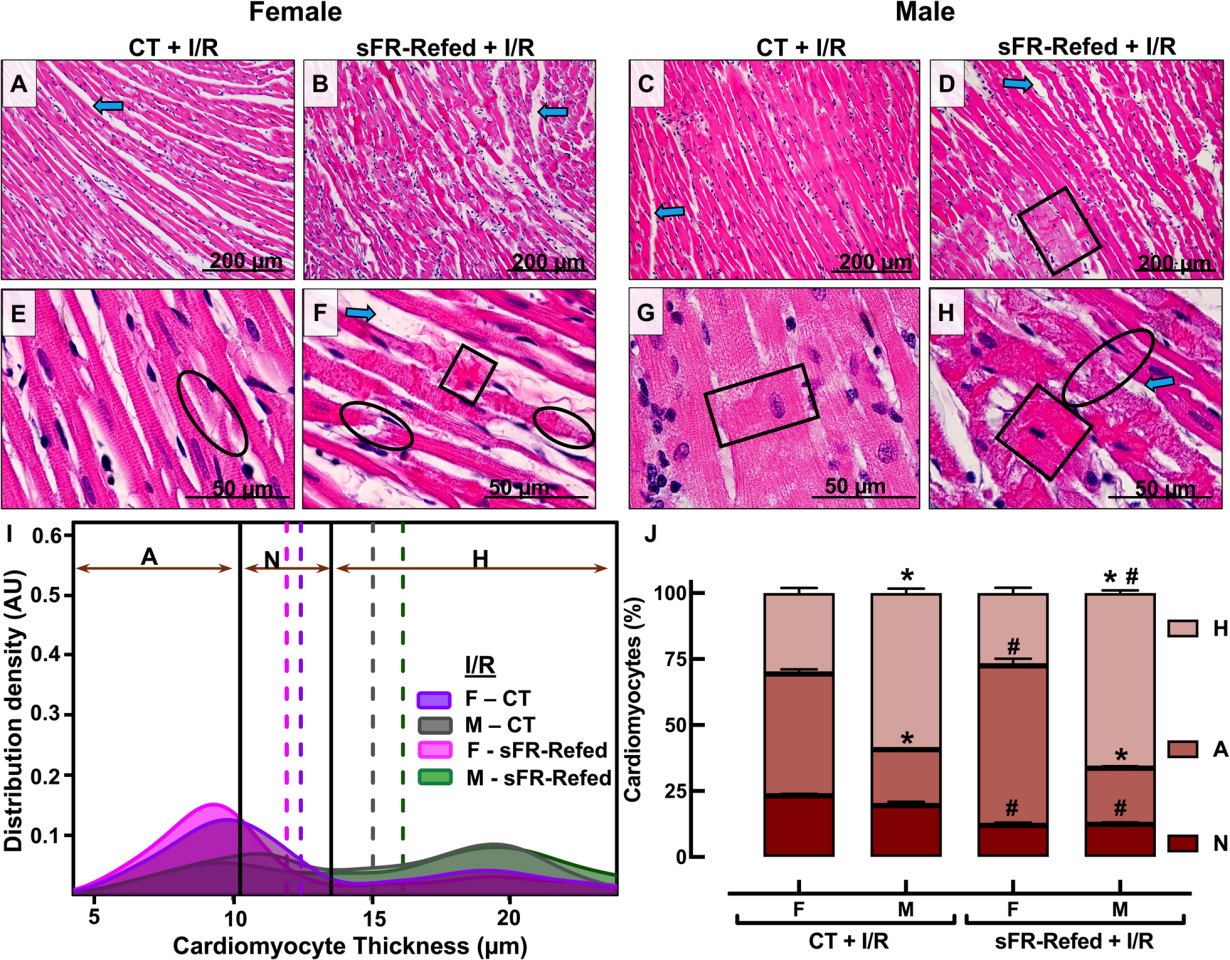
Effect of 3 months refeeding after severe food restriction followed by ischemia/reperfusion on cardiac pathology in the isolated heart. **A**–**H** Representative sections of the left ventricle of the heart after staining with hematoxylin and eosin (H&E) in female (F) and male (M) rats maintained on a normal diet for the duration of the study (CT) or for 3 months after a 2-week severe food restricted period (sFR-Refed) with subsequent exposure of the isolated heart to a 30 min period of ischemia followed by 60 min of reperfusion (I/R) at 200× (**A**–**D**) and 1000× (**E**–**H**) magnification. Images are representative of five rats per group. Greater interstitial space (blue arrow), fiber constrictions (black box) and focal cell lysis (black circle) are more prominent in sFR-Refed + I/R rats of both sexes compared to their same sex CT + I/R groups. **I** Gaussian Kernel density estimates of cardiomyocyte thickness from H&E-stained hearts. The color coded vertical dashed lines represent the mean in each animal group. **J** The percentage of cardiomyocytes below (A, atrophied) and above (H, hypertrophied) the cardiomyocyte diameter normal (N) range (depicted by two black vertical lines). Values are expressed as the mean ± SEM; ^#^*p* < 0.05 vs. CT, same sex and *p < 0.05 vs. F, same diet by two-way ANOVA and Bonferroni post-hoc test

Hearts from F-CT + I/R (Fig. 4A, E vs. 2A, E) and M-CT + I/R (Fig. 4C, G vs. 2C, G) rats exhibited significant damage after I/R. The disorganization of myofibers and dystrophic alterations of LV cardiomyocytes were pronounced. Multiple contractile lesions and focal lysis of cardiomyocytes were evident in CT + I/R rats from both sexes. The vast majority of cardiomyocytes from the CT + I/R group of either sex were not within the normal Gaussian distribution of cardiomyocyte thickness (Fig. 4I, J). Less than 25% of cardiomyocytes from the F-CT + I/R (23%) and M-CT + I/R (19%) groups had cell thicknesses within two standard deviations of the mean of the F-CT and M-CT cardiomyocytes, respectively (Fig. 2I, J). Furthermore, sex differences in the Gaussian distribution of cardiomyocyte size in the CT rats was evident after I/R (Fig. 4I, J). There was significantly more atrophy than hypertrophy in cardiomyocytes from F-CT + I/R rats, whereas the opposite was true for cardiomyocytes from the M-CT + I/R rats. This sex difference in cardiomyocyte size in response to I/R was amplified in the sFR-Refed groups. There were larger differences in the atrophy/hypertrophy ratio observed between female and male sFR-Refed rats after I/R.

### Effect of ischemia/reperfusion on collagen deposition in CT and sFR-Refed ats

**Fig. 5:**
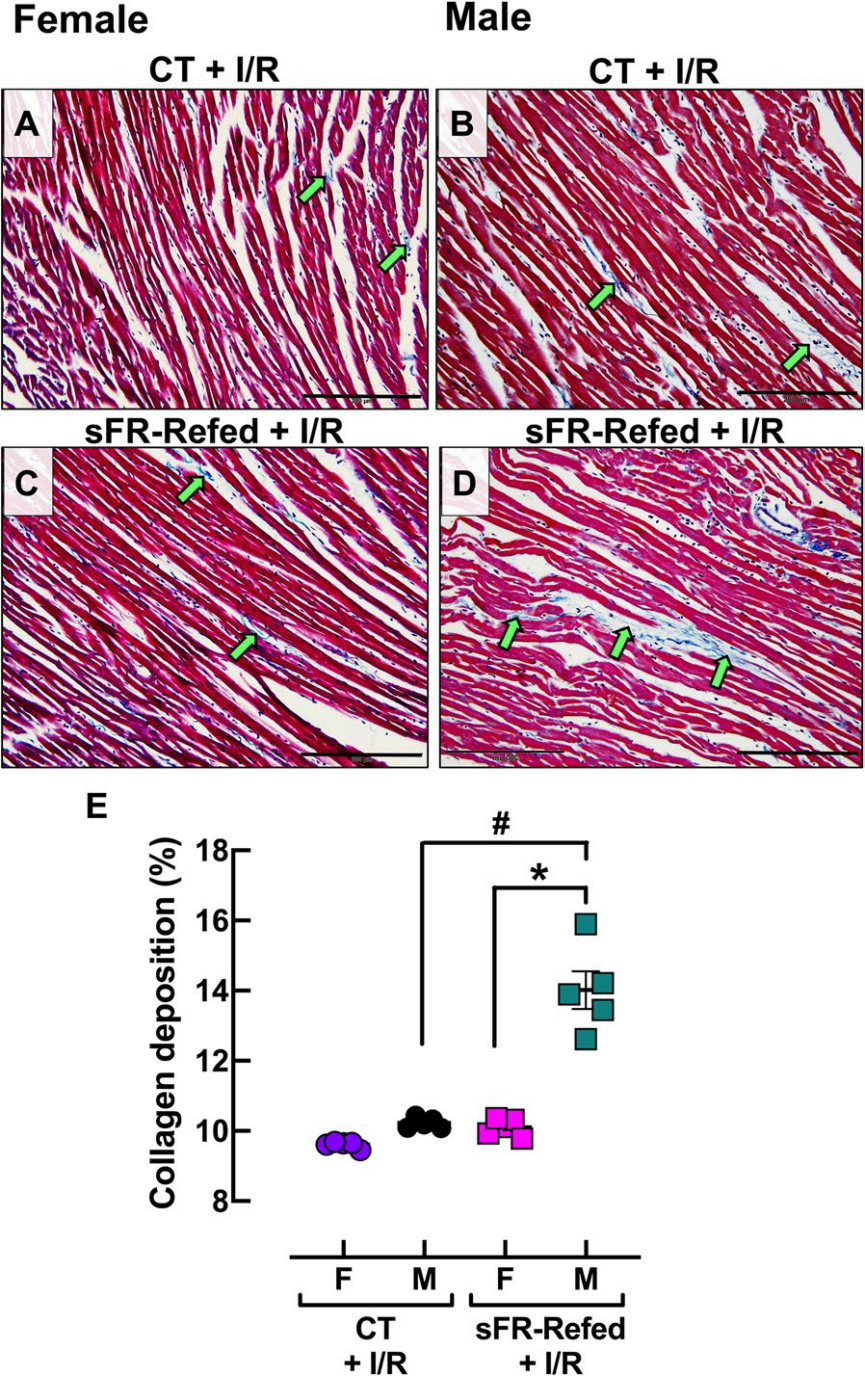
Effect of 3 months refeeding after severe food restriction followed by ischemia/reperfusion on collagen deposition in the isolated heart. **A**–**D** Representative sections of the left ventricle of the heart after staining with Masson’s trichrome in female (**A** and **C**) and male (**B** and **D**) rats maintained on a normal diet for the duration of the study (CT) or for 3 months after a 2-week severe food restricted period (sFR-Refed) with subsequent exposure of the isolated heart to a 30 min period of ischemia followed by 60 min of reperfusion (I/R) at 200× magnification. Images are representative of five rats per group. More collagen deposition (green arrows) is observed in male (M) compared to female (F)-sFR-Refed + I/R rats. **E** Quantitation of collagen deposition in the isolated heart of CT and sFR-Refed rats in both sexes. Values are expressed as the mean ± SEM; ^#^*p* < 0.05 vs. CT, same sex and *p < 0.05 vs. F, same diet by two-way ANOVA and Bonferroni post-hoc test

There were no sex differences in collagen deposition in hearts from CT-F + I/R (Fig. 5A, E) and CT-M + I/R (Fig. 5B, E) rats. While there was little difference in collagen deposition between the F-CT + I/R and F-sFR-Refed + I/R rats (Fig. 5E), the myocardium in M-sFR-Refed + I/R rats exhibited significantly more collagen deposition than the M-CT + I/R rats (Fig. 5E).

### Effects of refeeding after severe food restriction on perfusion pressure

**Fig. 6:**
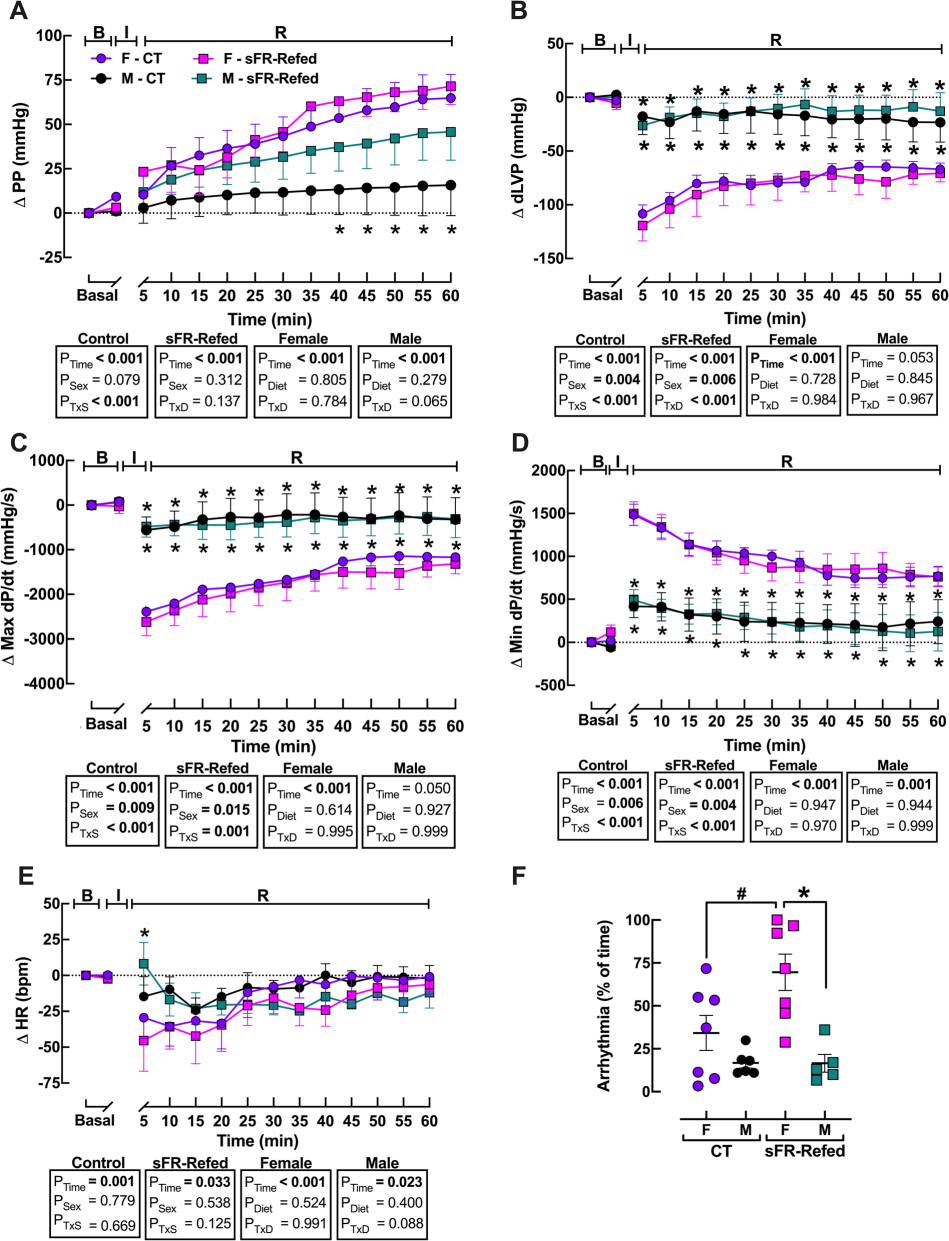
Effect of 3 months refeeding after severe food restriction on isolated heart function during reperfusion after ischemia. **A** Perfusion pressure (PP), **B** change in developed left-ventricular pressure (dLVP), **C** maximum rate of change in left ventricular pressure (Max d*P*/d*t*), **D** minimum rate of change in left ventricular pressure (Min d*P*/d*t*), **E** change in heart rate (HR), and, **F** percent of arrhythmias after 30 min of ischemia (I) in the basal state (B) and during the 60 min reperfusion (R) period in female (F, purple circle, red square; *n* = 7/group) and male (M, black circle, green square; *n* = 5/group) rats maintained on a normal diet for the duration of the study (CT, circle) or for 3 months after a 2-week sFR period (sFR-Refed, square). Values are expressed as the mean ± SEM; ^#^*p* < 0.05 vs. CT, same timepoint, same sex and *p < 0.05 vs. F, same diet, same timepoint by two-way ANOVA mixed-model for repeated measurements and Student’s *t* test for individual timepoints

Hearts from M-CT rats showed substantially lower baselines of perfusion pressure (PP) and maximum (Max) and minimum (Min) rates of rise of left ventricular pressure (d*P*/d*t*) compared to F-CT hearts (Table 3). Perfusion pressure was also substantially lower in the M-sFR-Refed rats compared to the F-sFR-Refed rats; however, this sex difference was not observed in the Max or Min d*P*/d*T* (Table 3). There were no differences in basal levels of left ventricular end diastolic pressure (LVEDP), developed left-ventricular pressure (dLVP) and heart rate (HR) within either sex between the CT and sFR-Refed rat groups (Table 3). Likewise, there were no differences within each sex between the CT and sFR-Refed groups in the change in dLVP (Fig. 6B) or in the max (Fig. 6C) or min (Fig. 6D) Δd*P*/d*t*; however, there was an effect of sex within the CT and sFR-Refed groups on these parameters. The ΔdLVP, ΔPP and ΔMax- and ΔMin-d*P*/d*t* during the reperfusion period were all larger in the female compared to the male rats. There was also no effect of sex on the HR response to reperfusion nor were there any differences observed between the CT and sFR-Refed rats (Fig. 6E).

**Table 3:** Effect of refeeding after severe food restriction on heart function

Parameter	Timepoint	F-CT	M-CT	F-sFR-Refed	M-sFR-Refed
PP mmHg	Baseline	142 ± 18	54.9 ± 6.3*	131 ± 6.1	52.3 ± 2.3*
	5 min	143 ± 12	56.8 ± 9.4*	151 ± 13	62.9 ± 6.5*
	60 min	197 ± 10	69.6 ± 15*	199 ± 11	96.8 ± 16*
LVEDP mmHg	Baseline	12 ± 1.5	12 ± 2.3	14 ± 1.5	11 ± 1.2
	5 min	78 ± 5.5	10 ± 2.0*	84 ± 11	19 ± 4.7*
	60 min	72 ± 6.8	12 ± 2.2*	67 ± 12	33 ± 11
dLVP mmHg	Baseline	140 ± 9.9	101 ± 12	141 ± 14	114 ± 18
	5 min	34.8 ± 10	81.2 ± 26*	26.8 ± 7.4	87.5 ± 15*
	60 min	76.3 ± 7.2	75.8 ± 26	75.3 ± 7.2	101 ± 26
Max d*P*/d*t* mmHg/s	Baseline	3083 ± 199	2098 ± 283*	3014 ± 289	2366 ± 352
	5 min	619.2 ± 217	1462 ± 421	418.8 ± 129	1819 ± 278*
	60 min	1837 ± 150	1699 ± 576	1724 ± 135	1991 ± 522
Min d*P*/d*t* mmHg/s (*n*)	Baseline	− 1768 ± 145	− 1178 ± 185*	− 1614 ± 152	− 1292 ± 244
	5 min	− 303.0 ± 83.4	− 703.5 ± 230	− 232.3 ± 61.5	− 757.2 ± 121*
	60 min	− 1017 ± 92.1	− 880.1 ± 326	− 970.8 ± 70.2	− 1122 ± 317
HR bpm	Baseline	178 ± 8.8	144 ± 19	145 ± 18	153 ± 5.8
	5 min	149 ± 14	140 ± 16	102 ± 18	163 ± 15*
	60 min	178 ± 5.9	143 ± 13*	141 ± 16	143 ± 7.2

Perfusion pressure (PP), left ventricle end diastolic pressure (LVEDP), developed left-ventricular pressure (dLVP), maximum and minimum left ventricular pressure (d*P*/d*t*) and heart rate (HR) in male (M, *n* = 5/group) and female (F, *n* = 7/group) rats maintained on a normal diet for the duration of the study (CT) or for 3 months after a 2-week severe food restricted period (sFR-Refed). Values are expressed as the mean ± SEM; *n*, group size; *p < 0.05 vs. F, same diet by unpaired Student’s *t* test

### Effect of refeeding after severe food restriction on cardiac arrhythmias

Cardiac arrhythmias were present in isolated hearts from both CT and sFR-refed rats during the reperfusion period (Fig. 6F). Whereas no differences were observed in the % arrhythmias between M-CT and M-sFR-Refed rats, isolated hearts from the F-sFR-Refed rats had twofold more arrhythmias than the F-CT rats during the reperfusion period (Fig. 6F).

## Discussion

The major finding of this study was that hearts from sFR-Refed rats of both sexes exhibited major cardiac damage 3 months after BW became indistinguishable from CT rats as a result of refeeding. However, notable sex differences were observed in the cardiac pathology in the sFR-Refed rats; the cardiomyocytes from the female rats exhibited more atrophy than hypertrophy, while the reverse was true in the males. The second major finding was that the F-sFR-Refed rats were more susceptibility to I/R-induced ventricular arrhythmias than the F-CT rats, whereas the frequency of ventricular arrhythmias in M-sFR-Refed rats were indistinguishable from the M-CT rats.

We previously showed that hearts from male rats subjected to 2 weeks of sFR exhibited major cardiac damage including myofibrillar disarray with contractions and cardiomyocyte lysis that was accentuated by I/R [4]. This current study extends our prior research by showing that cardiac damage is evident 3 months after BW has returned to CT levels as a result of refeeding in F-sFR-Refed rats (Figs. 2 and 4). The few clinical studies that have examined the long-term effects of prior exposure to very low food intake support the presence of cardiac injury long after the sFR period ended. Men who experienced the Siege of Leningrad had a higher incidence of mortality from ischemic heart disease and stroke 30 years later [10, 11]. In another study, survivors of a famine in China exhibited an increased risk for myocardial infarction, stroke and coronary heart disease 60 years later [12]. Thus, further investigation into the risk of developing cardiovascular disease in individuals who have experienced a sustained period of sFR earlier in life is warranted and could lead to therapeutics that would reverse this risk.

Our finding of hypertrophied cardiomyocytes in M-sFR-Refed rats 3 months after refeeding (Fig. 2) is supported by a study of cardiomyocytes from male rats subjected to sFR for 3 weeks followed by a 3-week period of refeeding [13]. This study showed the protein/DNA ratio was increased after the 3-week refeeding period, suggesting a cellular hypertrophic adaptative response occurred during the refeeding period.

While the literature is scarce when it comes to the effects of refeeding after sFR on histological changes to the male heart, even less is known regarding cardiac morphology in the female. Thus, of significant interest is the finding of sex differences in the type of cardiomyocyte damage induced by sFR. While the majority of cardiomyocytes in sFR-Refed rats of both sexes were no longer within the normal range of cardiomyocyte thickness, there were 1.8-fold as many atrophied cardiomyocytes in the F-sFR-Refed rats compared to the males, while there were 2.5-fold as many hypertrophied cardiomyocytes in M-sFR-Refed rats compared to the females (Fig. 2). In contrast, the atrophy present in F-sFR-Refed hearts suggest the cardiomyocytes underwent atrophic remodeling [14]. Mulroney et al. [15] showed that glomerular hypertrophy is observed in the remnant kidney of the uninephrectomized male rat but not in the female. The finding that more cardiomyocytes from M-sFR-Refed rats underwent hypertrophic remodeling than those from F-sFR-Refed rats is supported by the finding that there was less collagen deposition in the F-sFR-Refed rats compared to the M-sFR-Refed rats before (Fig. 3) or after I/R-induced injury (Fig. 5).

Fibroblasts are the primary cells responsible for the production of collagen, which plays a key role in maintaining the architecture of the heart and distributing the force generated by cardiomyocytes on the ventricular chamber [16]. We previously found M-sFR hearts had less collagen deposition than the M-CT hearts immediately after the 2-week sFR diet ended [4]. Three months after the start of refeeding, we now find M-sFR-Refed rats had significantly more collagen deposition than the M-CT rats, while F-sFR-Refed had less collagen deposition. Collagen synthesis and deposition in response to injury occurs over several weeks [17]. Thus, it is likely that collagen synthesis was triggered by sFR in the M rats; however, it took several months for collagen deposition to accumulate. Thus, hearts from M-sFR-Refed rats likely responded to the sFR by undergoing a remodeling process during refeeding.

Gonadal hormones could contribute to sex differences in cardiac remodeling. A study showed that female rat fibroblast cells isolated from hearts exposed to myocardial infarction or pressure overload exhibited less pro-fibrotic remodeling than cells from male rats [18]. Another study showed that 17β-estradiol protected ovariectomized mice from angiotensin II-induced cardiac hypertrophy and fibrosis [19]. Thus, estrogen could contribute to whether or not cardiomyocytes undergo atrophy or hypertrophy in response to sFR-induced cardiac injury months after refeeding. Testosterone may also be involved, since this sex hormone was shown to increase cardiomyocyte remodeling in response to myocardial infarction in both gonadectomized male and female mice [20]. Future studies on gonadal hormone regulation of cardiac pathology in sFR-Refed rats is, therefore, warranted.

Our previous studies on the acute effects of sFR showed that isolated hearts from M-sFR rats had higher PP and Min d*P*/d*t* and a marked decrease in dLVP and Max d*P*/d*t* [4]. Moreover, the M-sFR rats had nearly twice as many ventricular arrhythmias than the CT rats. Three months after refeeding, we now find no differences in the effect of I/R on PP, dLVP, d*P*/d*t*, HR and frequency of cardiac arrhythmias between the M-sFR-Refed and M-CT rats (Fig. 6). These findings of cardiac recovery in the male but not female sFR-Refed rat indicate that during the refeeding period male heart function was able to recover from the acute effects of sFR-induced injury. One contributing factor is likely related to differences in sex chromosomes and the organizational and activational effects of sex hormones [21, 22]. Testosterone exposure in utero*,* during development and after gonadal hormone maturity has profound growth promoting effects on muscle mass. Our observation that BW gain was threefold higher in the M-sFR-Refed compared to the F-sFR-Refed rats over the 3-month refeeding period emphasizes this point (Fig. 1A, Table 1). Thus, future studies investigating the impact of sex differences in growth rates and the accompanying physiological changes on cardiac function during recovery from sFR could lead to discovering novel therapeutic targets for reducing the dysfunction observed in F-sFR-Refed rats.

The observed sex differences in female susceptibility to I/R-induced cardiac arrhythmias is supported by other models of cardiac arrhythmias. Yan et al. showed that isolated hearts [23] and cultured myocytes [24] from female but not male rats developed ventricular arrhythmias in response to low doses of the endocrine disrupter, bisphenol A. Thus, estrogen may contribute to cardiac susceptibility to arrhythmias in females. Another factor in sex-specific susceptibility to I/R-induced cardiac arrhythmias is likely related to sex differences in heart function. Isolated hearts from female rats had higher PP and Min d*P*/d*t* and lower dLVP and Max d*P*/d*t* than the male hearts during the reperfusion period. Sex differences in the QT interval is another possible contributing factor. Longer QT intervals are associated with susceptibility to cardiac arrhythmias induced by drugs [25]. Men have shorter QT intervals than women in part as a result of testosterone, which has been shown to reduce the potassium current and the L-type calcium current [26]. It will be interesting in future studies to investigate the effects of sFR and refeeding on cardiac electrophysiology.

Clinical studies support these animal findings of female susceptibility to cardiac arrhythmias. A review of the Food and Drug Administration database showed women are more susceptible to drug-induced ventricular arrhythmias than men [25]. Previous studies of individuals on very low caloric diets have warned of dangerous cardiovascular consequences. The Food and Drug Administration and the Centers for Disease Control and Prevention discovered a pattern of sudden death due to intractable ventricular arrhythmias in individuals who had been dieting for prolonged periods of time and who had lost large amounts of weight [27]. A study by the National Heart Lung and Blood Institute of 17 of these dieters that died unexpectedly from intractable ventricular arrhythmias, suggested that rapid weight loss due to severe caloric restriction (300–400 kcal/day; 2.5 kg BW loss/week; 35% BW loss over 5 months) damaged the heart [27]. This conclusion is supported by case reports and small studies of patients on fasting diets that have reported sudden cardiac death and myofibrillar damage [28]. The chief finding was myocardial atrophy, which is consistent with protein calorie malnutrition in humans [29, 30] and monkeys [31]. What is of particular concern is the lasting cardiac damage in female rats that is evident 3 months after BW has returned to CT levels. This short period of severe calorie restriction is not uncommon among those who engage in very low-calorie dieting. Therefore, our findings justify the need for longitudinal studies of individuals who have experienced periods of inadequate caloric intake with malnutrition earlier in life for either voluntary or involuntary reasons.

### Study limitations

This study examined cardiac pathology and function after I/R in the isolated heart using the Langendorff technique to assess cardiac function and ventricular arrhythmias after I/R. Future studies are needed to investigate heart pathology and function under various ischemic conditions in the whole animal including in models of atrial fibrillation to expand our understanding of sex differences in cardiac responses to sFR and refeeding. Additional studies are also needed to address the impact of coronary flow on sex differences in the effects of sFR and refeeding, especially since sex differences in heart size and vessels likely modulate I/R-induced cardiac dysfunction. Further research is also needed to determine how age, diet and length and frequency of the sFR and refeeding periods impact the degree of cardiac damage and susceptibility to I/R-induced arrhythmias.

In conclusion, we found that a 15% reduction in BW over a 2-week sFR period in adult Fischer rats resulted in cardiomyocyte pathology 3 months after refeeding in both sexes; however, sex differences were evident in the type of cardiomyocyte injury. Cardiomyocytes in females exhibited more atrophy than hypertrophy, while the reverse was true in males. The sex-specific cardiac pathology in the female was associated with susceptibility to ventricular arrhythmias.

### Perspectives and significance

Our findings have ramifications for individuals who experience periods of sFR (e.g., 800–1000 kcal/day) [32, 33] due to voluntary (e.g., very low calorie dieting) or involuntary (very low food security) reasons. Furthermore, our study suggests women exposed to prior periods of sFR are at greater risk of developing ventricular arrhythmias and thus would benefit from more frequent screening for cardiovascular disease. Clearly, more research is needed to assess the impact of sFR on cardiovascular health and to develop interventions that would reduce the risk of developing cardiovascular disease later in life in both sexes.

## Data Availability

The data sets used and/or analyzed during the current study are available from the corresponding author on reasonable request.

## Abbreviations

BW: Body weight
CT: Control
dLVP: Developed left-ventricular pressure
d*P*/d*t*: Rate of change of left ventricular perfusion pressure
F: Female
H&E: Hematoxylin and eosin
HMI: Heart mass index
HR: Heart rate
I/R: Ischemia reperfusion
LV: Left ventricle of the heart
LVEDP: Left ventricle end diastolic pressure
M: Male
PP: Perfusion pressure
sFR: Severe food restriction
